# Biodistribution and Cellular Internalization of Inactivated SARS-CoV-2 in Wild-Type Mice

**DOI:** 10.3390/ijms23147609

**Published:** 2022-07-09

**Authors:** Anett Hudák, Gareth Morgan, Jaromir Bacovsky, Roland Patai, Tamás F. Polgár, Annamária Letoha, Aladar Pettko-Szandtner, Csaba Vizler, László Szilák, Tamás Letoha

**Affiliations:** 1 Pharmacoidea Ltd., H-6726 Szeged, Hungary; anett.hudak@pharmacoidea.eu (A.H.); labor@pharmacoidea.eu (L.S.); 2 Boeckeler Instruments, Inc., Tucson, AZ 85714, USA; gareth.morgan@boeckeler.com; 3 Delong Instruments a.s., 612 00 Brno, Czech Republic; jaromir.bacovsky@delong.cz; 4 Institute of Biophysics, Biological Research Centre, H-6726 Szeged, Hungary; patai.roland@brc.hu (R.P.); polgar.tamas.ferenc@gmail.com (T.F.P.); 5 Theoretical Medicine Doctoral School, Faculty of Medicine, University of Szeged, H-6720 Szeged, Hungary; 6 Department of Medicine, Albert Szent-Györgyi Clinical Center, Faculty of Medicine, University of Szeged, H-6720 Szeged, Hungary; letohadr@gmail.com; 7 Laboratory of Proteomics Research, Biological Research Centre, H-6726 Szeged, Hungary; aladar@brc.hu; 8 Institute of Biochemistry, Biological Research Centre, H-6726 Szeged, Hungary; vizler.csaba@brc.hu

**Keywords:** SARS-CoV-2, cellular uptake, heparan sulfate proteoglycans, syndecans, mouse

## Abstract

Despite the growing list of identified SARS-CoV-2 receptors, the human angiotensin-converting enzyme 2 (ACE2) is still viewed as the main cell entry receptor mediating SARS-CoV-2 internalization. It has been reported that wild-type mice, like other rodent species of the Muridae family, cannot be infected with SARS-CoV-2 due to differences in their ACE2 receptors. On the other hand, the consensus heparin-binding motif of SARS-CoV-2’s spike protein, PRRAR, enables the attachment to rodent heparan sulfate proteoglycans (HSPGs), including syndecans, a transmembrane HSPG family with a well-established role in clathrin- and caveolin-independent endocytosis. As mammalian syndecans possess a relatively conserved structure, we analyzed the cellular uptake of inactivated SARS-CoV-2 particles in in vitro and in vivo mice models. Cellular studies revealed efficient uptake into murine cell lines with established syndecan-4 expression. After intravenous administration, inactivated SARS-CoV-2 was taken up by several organs in vivo and could also be detected in the brain. Internalized by various tissues, inactivated SARS-CoV-2 raised tissue TNF-α levels, especially in the heart, reflecting the onset of inflammation. Our studies on in vitro and in vivo mice models thus shed light on unknown details of SARS-CoV-2 internalization and help broaden the understanding of the molecular interactions of SARS-CoV-2.

## 1. Introduction

Severe acute respiratory syndrome coronavirus 2 (SARS-CoV-2) presents formidable challenges for clinicians and researchers worldwide [1,2]. Growing details on the molecular background of SARS-CoV-2 infection enabled the development of efficient vaccines and novel antivirals [3,4]. Despite the growing list of identified SARS-CoV-2 receptors, the human angiotensin-converting enzyme 2 (ACE2) is still viewed as the major cell entry receptor during SARS-CoV-2 infection [5,6]. However, recent studies show SARS-CoV-2’s ACE2-independent cellular entry [7,8,9]. Due to differences in their ACE2 receptors, wild-type (WT) mice reportedly cannot be infected with SARS-CoV-2 [10,11]. On the other hand, the evolutionary conserved heparin-binding motif in SARS-CoV-2’s spike protein, PRRAR, enables the attachment to mammalian heparan sulfate proteoglycans (HSPGs), other well-established SARS-CoV-2 receptors [12,13,14,15,16,17].

Previously, we explored the contribution of syndecans (SDCs), an evolutionary conserved transmembrane HSPG family with a well-established role in clathrin- and caveolin-independent endocytosis, to the cellular internalization of SARS-CoV-2 [18,19,20,21,22,23]. According to our findings, SDCs mediate the cellular entry of SARS-CoV-2 by attaching its S1 subunit containing the heparin-binding motif (PRRAR). Among SDCs, syndecan-4 (SDC4), the isoform enriched in the lung, mediated the cellular uptake of SARS-CoV-2 and its Delta variant most efficiently in [18,24]. Competitive inhibition with heparin or heparin-binding peptides reduced the cellular entry of SARS-CoV-2, demonstrating the importance of attachment to heparan sulfate (HS).

HS, a sulfated polysaccharide belonging to the family of glycosaminoglycans (GAGs), is ubiquitously distributed on the surfaces of animal cells and in the extracellular matrix [25,26]. Protein–GAG interactions play a prominent role in cell–cell interactions, cell growth, and viral infections [27,28,29]. The consensus heparin/HS-binding motifs XBBXBX or XBBBXXBX (B being the basic amino acids arginine, lysine, or histidine and X being one of a range of aliphatic/aromatic amino acids) endow proteins to attach to HS [30,31]. Due to their conserved structures, murine SDCs are highly identical to their human counterparts. Besides their ectodomains being 70% identical, all putative glycosaminoglycan attachment sites are identical. Furthermore, the transmembrane domain is 96% identical, while the cytoplasmic domain is 100% identical, including three identically located tyrosine residues [32]. Despite the subtle differences in the sulfation pattern of mammalian HSPGs, the consensus HS-binding motifs, like the one present in SARS-CoV-2’s spike, enable the attachment of heparin-binding proteins to mammalian HSPGs, including SDCs [17,25,33]. During SDC-mediated endocytosis, attachment of ligands triggers the clustering of SDCs and stimulates the internalization of the SDC–ligand complex [20,34,35].

Given the affinity of the HS-binding motif of SARS-CoV-2’s spike towards HS and the established role of HSPGs in nonclassical endocytosis, we decided to analyze the cellular uptake of inactivated SARS-CoV-2 particles in in vitro and in vivo mice models. Cellular and animal studies enabled the focused analyses of SARS-CoV-2 cellular entry and revealed novel aspects of SARS-CoV-2 internalization.

## 2. Results

### 2.1. Electron Microscopy of SARS-CoV-2’s Internalization in Murine Cell Lines

**Figure 1:**
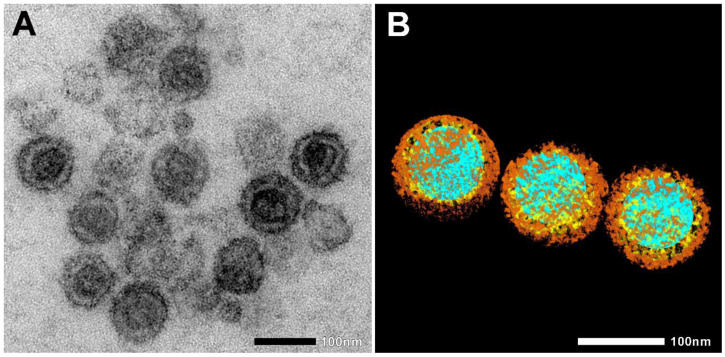
Electron microscopic images of heat-inactivated SARS-CoV-2. (**A**) Native electron microscopic image. (**B**) For the visualization of SARS-CoV-2 morphology, regions of interest, such as nucleic acid (blue), nucleocapsid (yellow), and spike proteins (brown), were segmented manually. Scale bar 100 nm.

Heat-inactivated SARS-CoV-2 (strain 2019-nCoV/USA-WA1/2020; see Figure 1A,B) was incubated with two murine cell lines—L929 fibroblasts and RAW 264.7 (RAW) macrophages—for 10, 30, and 180 min at 37 °C. The cells were then fixed, and cellular internalization was analyzed with electron microscopy (EM), utilizing two systems: JEOL JEM-1400Flash and Delong LVEM 25.

**Figure 2:**
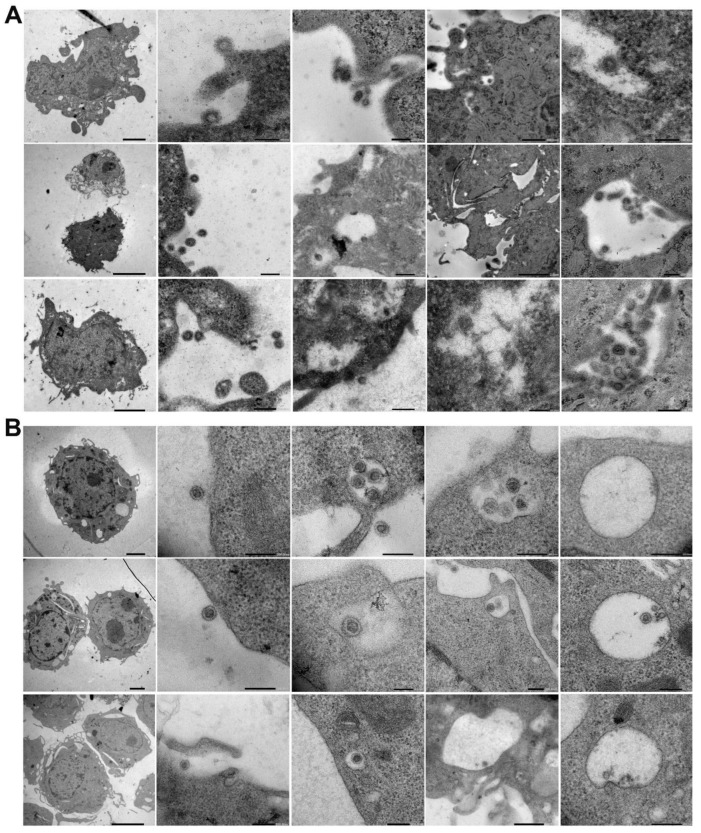
Electron microscopic visualization of SARS-CoV-2 internalization into murine cell lines. L929 (**A**) and RAW (**B**) cells were incubated with heat-inactivated SARS-CoV-2 for various amounts of time (10, 30, or 180 min) at 37 °C. After incubation, the cells were washed and fixed, and SARS-CoV-2 internalization was analyzed with a JEOL JEM-1400Flash electron microscope. Representative images of three independent experiments are shown.

**Figure 3:**
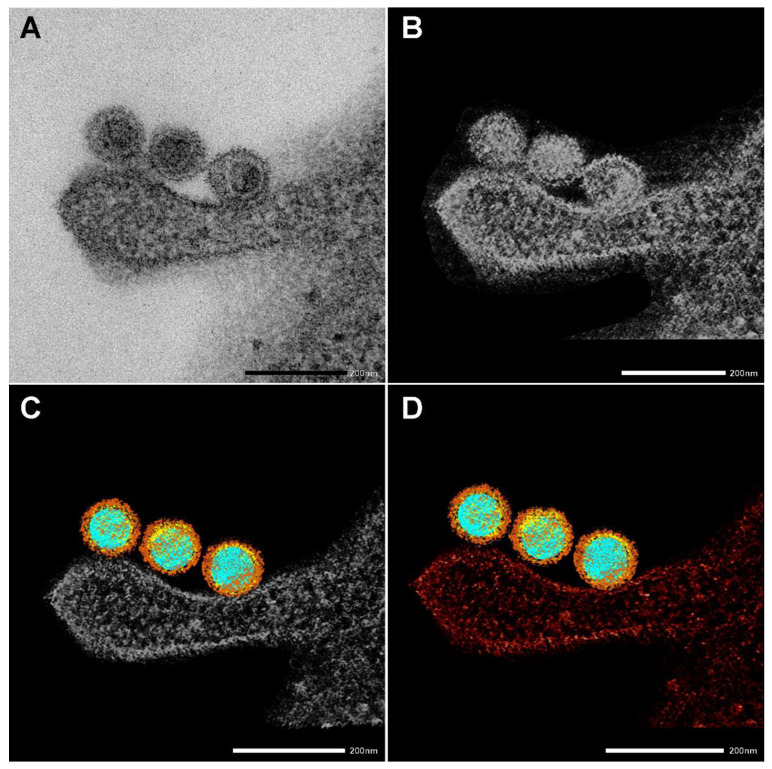
Creating a 3D electron tomography. (**A**) SARS-CoV-2 particles bound by an invading RAW macrophagen at 10 min of incubation at 37 °C. Scale bar 200 nm. (**B**–**D**) The images presented are utilized to create a 3D electron tomography. Regions of interest, such as nucleic acid (blue), nucleocapsid (yellow) spike proteins (brown), and the neighboring macrophage (red), were segmented manually.

EM nicely revealed the progress of SARS-CoV-2’s endocytosis in L929 and RAW cells from surface attachment to endosome engulfment (Figure 2A,B). Thus, EM showed that despite the differences in murine ACE2, inactivated SARS-CoV-2 particles can be bound and internalized by murine cells (Figure 2 and Figure 3 and Supplementary Figure S1).

EM also nicely depicted the SARS-CoV-2 morphology and the virus’s interaction with cellular components. Figure 3 shows the four stages of creating an electron tomography of the viruses’ attachment to an invading RAW macrophage (the resulting 3D tomogram is shown in the Graphical Abstract). Individual virus particles with their characteristic morphological features can be observed where different colors represent the various regions. Regions of interest, such as nucleic acid (blue), nucleocapsid (yellow), and spike proteins (brown), were segmented manually. Furthermore, the neighboring RAW macrophage was also segmented and represented with red color.

### 2.2. Flow Cytometric Assessment of SARS-CoV-2’s Internalization in Murine Cell Lines

**Figure 4:**
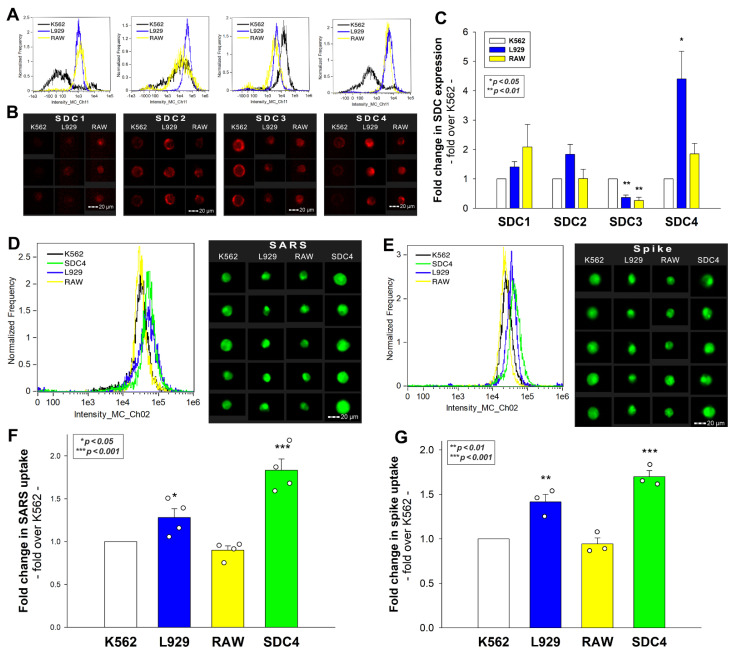
Flow cytometric assessment of SDC expression and SARS-CoV-2’s and its spike protein’s uptake in human and murine cell lines. SDC expression of L929, RAW, and K562 cells was analyzed with imaging flow cytometry using fluorescently labeled SDC antibodies. (**A**,**B**) Representative flow cytometry histograms and fluorescent images showing SDC expression levels in L929, RAW, and K562 cells. Scale bar = 20 μm. (**C**) Detected SDC expression level cells were normalized to K562s. The bars represent the mean + SEM of three independent experiments. Statistical significance was assessed with analysis of variance (ANOVA). * *p* < 0.01; ** *p* < 0.001. (**D**–**G**) L929, RAW, and K562 cells and SDC4 transfectants (created in K562 cells) were incubated with either inactivated SARS-CoV-2 (at 1 MOI) or spike protein (50 nM) for 3 h at 37 °C. After incubation, the cells were washed, trypsinized, fixed, permeabilized, and treated with antibodies specific for the spike glycoprotein (and AF 488-labeled secondary antibodies). Cellular uptake of SARS-CoV-2 and spike was then analyzed with imaging flow cytometry. (**D**,**E**) Representative flow cytometry histograms and fluorescent images showing the intracellular fluorescence of SARS-CoV-2- and spike-treated cells. Scale bar = 20 μm. (**F**,**G**) Detected fluorescence intensities were normalized to SARS-CoV-2- or spike-treated K562 cells as standards. The bars represent the mean + SEM of four (**F**) and three (**G**) independent experiments. Statistical significance vs. standards was assessed with ANOVA. * *p* < 0.05; ** *p* < 0.01; *** *p* < 0.001.

Previously, we showed that SDCs, especially SDC4 enriched in the lung, mediate the cellular internalization of SARS-CoV-2 in human cell lines. After revealing the internalization of inactivated SARS-CoV-2 into murine cell lines with EM, we assessed the SDC expression of L929 and RAW cells. As the K562 human cell line reportedly lacks HSPGs except for shallow amounts of endogenous betaglycan and SDC3, K562 cells were chosen as standards [33,34,35]. Compared with K562, L929 and RAW cells exhibited slightly increased SDC1 yet significantly decreased SDC3 expression (Figure 3A–C). Compared with K562, L929 cells showed higher SDC2 expression, although the increase was not statistically significant (*p* > 0.05). SDC4 expression of RAW and L929 was significantly higher than that of K562, but the increase (~4.5-fold) was more definite in L929 cells. Cellular internalization of inactivated SARS-CoV-2 in RAW cells was slightly lower than in K562, yet L929 fibroblasts—whose SDC4 expression was highest among the three lines—exhibited significantly increased SARS-CoV-2 uptake (Figure 4D–F). Compared with stable SDC4 transfectants (created in K562 cells) with ~20-fold SDC4 expression (Supplementary Figure S2), the cellular uptake of SARS-CoV-2 in L929 cells was significantly lower (Figure 4D–F). Cellular internalization of the spike protein (incubated at a concentration of 50 nM for 3 h at 37 °C) exhibited a pattern similar to the heat-inactivated SARS-CoV-2 particles (Figure 4E,G).

Incubating the cells with SARS-CoV-2 at 4 °C, a temperature ceasing endocytosis due to rigid cellular membranes, gave very low cellular fluorescence (Supplementary Figure S3), thus validating the reliability of the intracellular signals detected on SARS-CoV-2-treated cells at 37 °C. It is also worth noting that SARS-CoV-2 or spike protein treatment did not affect cell viability (Supplementary Figure S4).

### 2.3. Effect of SDC4 Knockdown on Virus Internalization

**Figure 5:**
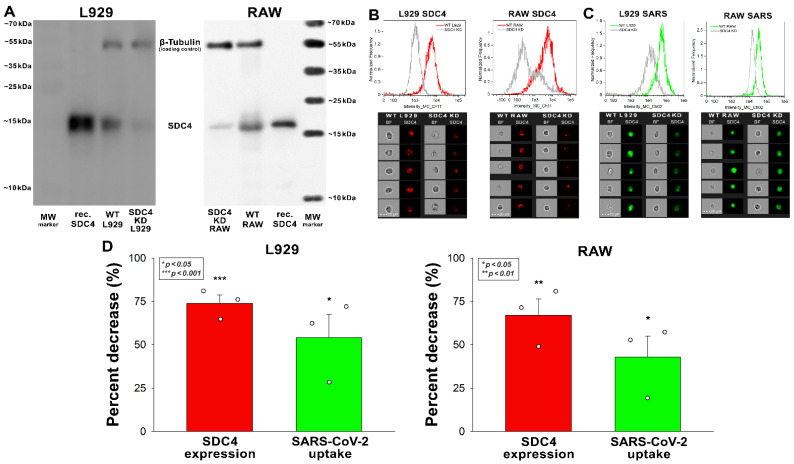
Effect of SDC4 knockdown (KD) on the internalization of inactivated SARS-CoV-2 into L929 and RAW cells. SDC4 KD in L929 and RAW cells was performed using a lentiviral vector specific to mouse SDC4. (**A**) Western blot validation of SDC4 kD in L929 and RAW cells. β-tubulin was used as a loading control. (**B**) SDC4 expression levels were also measured with imaging flow cytometry, as shown by the representative histograms and cellular images. Scale bar = 20 μm. Detected SDC4 levels of KD cells were normalized to WT cells as standards. (**C**,**D**) WT and SDC4 KD cells were incubated with inactivated SARS-CoV-2 (1 MOI) for 3 h at 37 °C. After incubation, the cells were washed, trypsinized, fixed, permeabilized, and treated with antibodies specific for the spike glycoprotein (and AF 488-labeled secondary antibodies). Cellular uptake of SARS-CoV-2 and spike was then analyzed with imaging flow cytometry. (**C**) Representative flow cytometry histograms and cellular images show the intracellular fluorescence of SARS-CoV-2-treated WT or SDC4 KD cells. Scale bar = 20 μm. (**D**) The effect of SDC4 KD on SDC4 expression and SARS-CoV-2 uptake expressed as percent decrease. The bars represent the mean + SEM of three independent experiments. Statistical significance vs. controls was assessed with ANOVA. * *p* < 0.05; ** *p* < 0.01; *** *p* < 0.001.

Reducing the SDC4 expression of L929 and RAW cells with knockdown (KD) reduced the cellular uptake of the virus (Figure 4A–D). That is, a ~70% decrease in SDC4 expression resulted in a ~50% reduction in SARS-CoV-2 uptake in the applied murine cell lines (Figure 5B–D).

### 2.4. Confocal Microscopic Assessment of SARS-CoV-2’s Internalization in Murine Cell Lines

**Figure 6:**
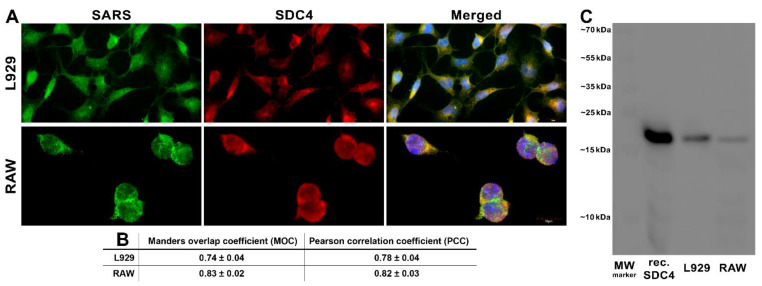
SARS-CoV-2 colocalizes with SDC4 during cellular entry. L929 and RAW cells were incubated with heat-inactivated SARS-CoV-2 (at 1 MOI) for 3 h at 37 °C. After incubation, the cells were washed, fixed, permeabilized, and treated with antibodies specific for the spike glycoprotein (AF 488 labeled) and SDC4 (APC labeled). Colocalization of SARS-CoV-2 with SDCs was analyzed with confocal microscopy. (**A**) Microscopic analyses of SARS-CoV-2 and SDC colocalization. Representative images of three independent experiments are shown. Scale bar = 10 μm. (**B**) The MOC and PCC ± SEM for the overlap and colocalization of SDC with SARS-CoV-2 (indicated below the images) were calculated by analyzing 18 images with an average of 12 cells in each image (from 3 separate samples). Scale bar = 10 μm. (**C**) A representative Western blot showing SDC4 immunoprecipitated with SARS-CoV-2 in L929 and RAW cells. Lane 1: 0.5 µg of SDC4; lanes 2–3: immunoprecipitates of SARS-CoV-2-treated L929 and RAW cells. Standard protein size markers are indicated on the right.

Next, we explored the colocalization of SARS-CoV-2 with SDC4 during uptake into L929 and RAW cells (Figure 6A,B). Taken at 3 h of incubation with inactivated SARS-CoV-2, confocal microscopic studies revealed a high degree of overlap (expressed with the Manders overlap coefficient (MOC) in Figure 6A,B) and excellent correlation (see Figure 6B’s Pearson correlation coefficient (PCC)) between SARS-CoV-2 and SDC4 in both cell lines (Figure 6A,B). Co-immunoprecipitation also confirmed that the inactivated SARS-CoV-2 binds to SDC4 of L929 and RAW cells (Figure 6C).

Cells incubated with inactivated SARS-CoV-2 at 4 °C—a temperature when endocytosis stops due to the rigidity of cellular membranes—exhibited relatively (i.e., compared with cells incubated at 37 °C) low surface-based signals, yet SARS-CoV-2 still showed significant overlap (MCC) and correlation (PCC) with SDC4 (Supplementary Figure S5). AF 488-labeled goat anti-mouse IgG—the secondary antibody used for SARS-CoV-2 detection—and APC-labeled SDC4 antibodies in the absence of the virus showed very low green autofluorescence (Supplementary Figure S6), hence validating the high intracellular signals detected on the SARS-CoV-2-treated cells at 37 °C.

### 2.5. In Vivo Biodistribution of Inactivated SARS-CoV-2 in Mice

**Figure 7:**
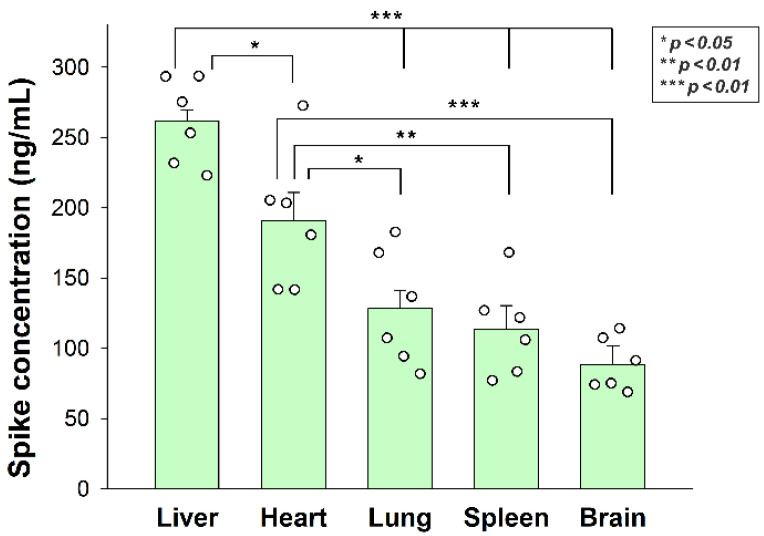
In vivo internalization of inactivated SARS-CoV-2 in mice. C57BL/6 mice were treated with heat-inactivated SARS-CoV-2. After 3 h of incubation, the mice were euthanized, various organs were collected, and their SARS-CoV-2 spike concentration was measured with a spike-specific ELISA kit. Each group contained 6 animals. The bars represent the mean + SEM. Statistical significance was assessed with ANOVA. * *p* < 0.05; ** *p* < 0.01; *** *p* < 0.001.

After assessing the in vitro cellular uptake of inactivated SARS-CoV-2, we analyzed its in vivo biodistribution in WT mice. Inactivated SARS-CoV-2 particles were injected intravenously (i.v.) into 12-month-old C57BL/6 mice. After 3 h of incubation, the mice were anesthetized, and blood was collected with cardiac puncture. After transcardial perfusion with ice-cold PBS (2 mL/min, 8 min), the brain, the heart, the liver, the lung, and the spleen—organs validated for their SDC4 expression by the Human Protein Atlas—were removed and frozen in dry ice for further examinations [36,37,38]. According to the Human Protein Atlas, SDC4 expression is highest in the liver, the lung, and the brain, while the heart muscle shows medium and the spleen exhibits modest SDC4 expression (Supplementary Table S1). The extent of SARS-CoV-2 internalization—as measured with a SARS-CoV-2 spike-specific ELISA—reflected not just the route that i.v. administered particles follow after administration, but also the SDC4 tissue expression. As shown in Figure 7, significantly more SARS-CoV-2 particles were taken up by the liver than the other tissues. The heart also accumulated more SARS-CoV-2 particles than the lung, the spleen, and the brain. The brain internalized the heat-inactivated viral particles the least among the investigated organs. However, the difference of the measured spike concentrations between the lung and the brain was not significant, showing the substantial amount of SARS-CoV-2 entering the brain (Figure 7). Spike concentrations in the organs of untreated controls were marginal and about in the range of the standard error of the means of the SARS-CoV-2-treated animals (Supplementary Figure S7).

**Figure 8:**
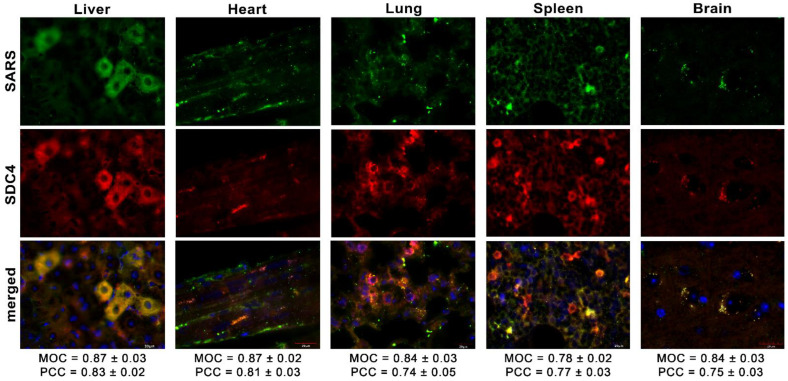
Colocalization of SDC4 with SARS-CoV-2 in various organs of the mice. Liver, heart, lung, spleen, and brain samples of mice receiving i.v. administration of inactivated SARS-CoV-2. Tissue SDC4 is detected with an APC-labeled SDC4 antibody, while SARS-CoV-2 with an AF 488-labeled spike antibody. Scale bar = 20 μm. MOC ± SEM and PCC ± SEM for the overlap and colocalization of SARS-CoV-2 with SDC4 were calculated by analyzing 18 images from 3 samples of each animal.

Simultaneously, we explored the in vivo internalization of inactivated SARS-CoV-2 with confocal microscopy. These microscopic analyses revealed a high overlap (see MOCs in Figure 8) and an excellent correlation between the internalized SARS-CoV-2 and SDC4. Contrary to SARS-CoV-2-treated animals, histochemistry did not reveal the presence of SARS-CoV-2 in control animals not receiving SARS-CoV-2 treatment (Supplementary Figure S8).

### 2.6. Inactivated SARS-CoV-2 Raises Tissue TNF-α Concentrations

**Figure 9:**
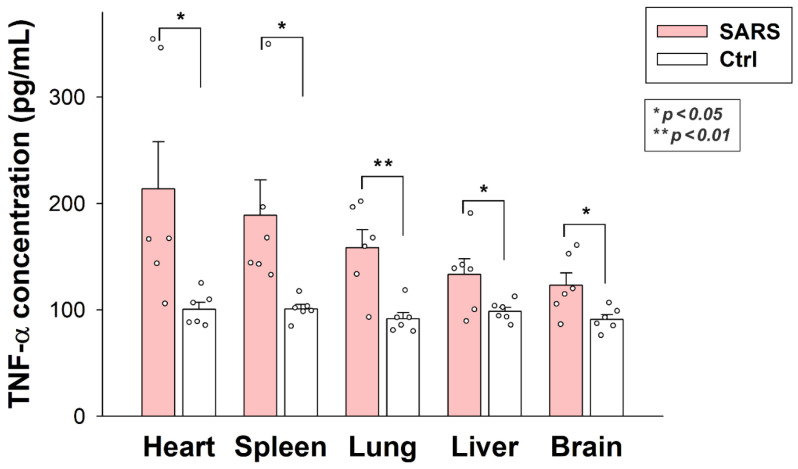
Inactivated SARS-CoV-2 raises TNF-α concentrations in various organs. TNF-α concentrations of the brain, heart, liver, lung, and spleen extracts of SARS-CoV-2-treated and untreated mice were measured with a mouse TNF-α ELISA kit. Each group contained 6 animals. The bars represent the mean + SEM. Statistical significance vs. untreated controls was assessed with ANOVA. * *p* < 0.05; ** *p* < 0.01.

As revealed by TNF-α-specific ELISA analyses, internalized SARS-CoV-2 particles significantly raised tissue TNF-α concentrations in all organs (brain, heart, liver, lung, and spleen). As shown in Figure 9, inactivated virus particles triggered the highest TNF-α concentrations in the heart, followed by the lung, spleen, liver, and brain. Thus, inactivated SARS-CoV-2 triggers proinflammatory pathways in all organs it enters. SARS-CoV-2-triggered tissue inflammation is related to the number of viral particles accumulated by the specific tissue, except for the liver, which gathered the most significant number of viral particles. However, the liver’s TNF-α levels were similar to the brain’s.

## 3. Discussion

More than two and a half years after the emergence of SARS-CoV-2, there are still many unanswered questions surrounding the cellular biology of SARS-CoV-2 [39,40]. One of the most significant challenges of COVID-19 pathomechanism is understanding the contribution of SARS-CoV-2’s cellular entry to infection [41]. For that, the exclusivity of ACE2 in the cellular internalization of SARS-CoV-2 needs to be challenged. This is especially true when growing scientific evidence supports SARS-CoV-2’s ACE2-independent cellular entry [7,8,9]. We have been exploring the SDC-dependent entry of SARS-CoV-2 and its Delta variant. According to our studies, SDCs—especially SDC4, the isoform enriched in the lung—facilitate the internalization of SARS-CoV-2 and the Delta variant by attaching their spike proteins [18,24]. Decreasing SDC expression exerted a more significant reduction in the cellular uptake of SARS-CoV-2 than ACE2 [18,24]. Our previous studies also showed that ACE2 activity is not required for the efficient cellular translocation of the virus [18,24]. That is, ACE2 inhibition had a minor effect on the cellular uptake of SARS-CoV-2 and none on its Delta variant, suggesting that ACE2 serves as a docking site rather than an endocytosis receptor driving the membrane engulfment and the internalization of the virus [24]. Besides SDCs, other cellular SARS-CoV-2 receptors have been identified, enabling the ACE2-independent cellular uptake of the virus [7,8,9].

Due to differences between human and murine ACE2, WT mice, like other rodent species of the Muridae family, reportedly cannot be infected with SARS-CoV-2 [42]. However, mice still express HSPGs, including SDCs, enabling the attachment and efficient internalization of specific ligands, including viruses and other parasites [43,44]. SARS-CoV-2’s spike has a high affinity towards HS due to its consensus heparin-binding motif (PRRAR) [13,16,18,24]. The same motif enables human fibronectin attachment to rat HSPGs, including SDCs and glypicans, despite the minor structural differences between rat and human HSPGs [17].

Considering the presence of the evolutionary conserved heparin-binding motif in SARS-CoV-2’s spike and the involvement of mammalian HSPGs in the cellular uptake of ligands, we conducted a series of in vitro and in vivo studies on the internalization of inactivated SARS-CoV-2 particles. Our results show the efficient internalization of inactivated SARS-CoV-2 particles into murine cell lines with established SDC4 expression. Inactivated SARS-CoV-2 particles colocalize with SDC4 during cellular uptake, suggesting a common entry route. After i.v. administration in vivo, the virus particles are taken up by various organs of the mice, including the liver, the heart, the lung, the spleen, and the brain. Internalized SARS-CoV-2 particles exhibit excellent overlap with tissue SDC4, confirming the role of SDC4 in mediating the in vivo cellular uptake of the virus. The emergence of the viral particles in the brain is also worth emphasizing. SDCs have been known to facilitate HIV-1 invasion into the brain [45]. Thus, even in the case of structurally incompetent ACE2, the existing cellular entry routes offer efficient SARS-CoV-2 transport into the brain. After entering the organs, the internalized SARS-CoV-2 particles raise tissue TNF-α concentrations, suggesting the onset of local inflammation. Thus, immune mechanisms readily detect the foreign virus particles in WT mice.

Thus, our data confirm previous results on the SDC4-mediated cellular entry of SARS-CoV-2, suggesting that even in the case of a structurally divergent murine ACE2, SDC4—a receptor with well-established endocytic functions—still manages to facilitate cellular uptake of the virus. Considering that the same heparin-binding motif present in SARS-CoV-2’s spike endows fibronectin to attach to rodent SDCs, the cellular entry of SARS-CoV-2 to murine cells expressing SDCs, especially SDC4, is not a surprise. Several endocytic mechanisms of SARS-CoV-2 have been exposed [46,47]. Among them, clathrin-mediated endocytosis was suggested to be a key aspect of virus infectivity [48]. SARS-CoV-2, just like other β-coronaviruses, uses lysosomes for egress, hence demonstrating the importance of the endosomal/lysosomal system to SARS-CoV-2’s life cycle and infection [48,49,50,51]. SDC-mediated cellular entry occurs mainly through clathrin-independent routes involving lipid rafts [52]. Ligands entering the cells via clathrin-independent SDC-mediated pathways can bypass lysosomal degradation [52,53,54,55,56]. Thus, by avoiding lysosomes, the SDC-mediated internalization of SARS-CoV-2 can paradoxically inhibit the formation of new viruses egressing from lysosomes. As β-coronaviruses hijack the endolysosomal system to ensure infection, endocytic pathways avoiding lysosomal entry offer a less favorable milieu for SARS-CoV-2 replication. The viral cycle involves six phases: (1) host cell binding, (2) host cell entry, (3) uncoating, (4) replication, (5) assembly, and (6) release from the cell [51]. Even if cell binding and entry are effective, deficient viral replication, assembly, and release can undermine the efficacy of infection. As SDC-mediated endocytosis, unlike the classical clathrin-mediated pathways, rather avoids lysosomal entry, the efficient cellular uptake of the inactivated virus does not contradict the lack of efficient SARS-CoV-2 infection in WT mice. On the other hand, even in the case of inefficient replication, the viral particles can still trigger inflammatory pathways in WT mice, a clear sign of immune recognition of the foreign viral particles.

In summary, our data show the efficient entry of SARS-CoV-2 particles in WT mice and confirm recently explored evidence on the ACE2-independent entry routes of SARS-CoV-2. The obtained evidence also supports the heavy influence of endocytic pathways on virus infection and replication. Thus, our current manuscript provides several critical findings on SARS-CoV-2 to better understand the virus’s cellular biology.

## 4. Materials and Methods

### 4.1. Heat-Inactivated SARS-CoV-2

Heat-inactivated SARS-CoV-2 (strain: 2019-nCoV/USA-WA1/2020) was purchased from ATCC (Manassas, VA, USA; cat. no. ATCC VR-1986HK).

### 4.2. SDC4 Constructs and Transfection, Cell Cultures

SDC4 transfectants, established in K562 cells (ATCC, Manassas, VA, USA; cat. no. CCL-243), were created as described previously [18]. Stable SDC4 transfectants were selected by measuring SDC4 expression with flow cytometry using APC-labeled SDC4 antibodies (R&D Systems, Minneapolis, MN, USA; SDC4: monoclonal rat IgG2a clone #336304, cat. no. FAB29181A [20,54]), along with the respective isotype controls (R&D Systems; rat IgG2a APC isotype control, cat. no. IC006A).

L929 (Merck KGaA, Darmstadt, Germany; cat. no. 85011425-1VL) and RAW 264.7 cells (ATCC, Manassas, VA, USA, cat. no. TIB-71) were grown in DMEM, 10% FBS at 37 °C, 5% CO_2_.

### 4.3. Electron Microscopy of SARS-CoV-2 Cellular Attachment and Internalization

L929 and RAW cells were grown on glass-bottom 35 mm culture dishes. After 24 h, the cells were incubated with SARS-CoV-2 (1 MOI) for various amounts of time (10, 30, and 180 min) in DMEM/F12 medium (supplemented with 10% FBS) at 37 °C. After incubation with or without SARS-CoV-2, the cells were rinsed two times with ice-cold PBS, fixed in a Karnovsky solution containing 2% paraformaldehyde (Sigma; St. Louis, MO, USA) and 2.5% glutaraldehyde (Polysciences; Warrington, PA, USA) in phosphate buffer overnight at 4 °C [57,58]. Then the samples were rinsed in distilled water (pH 7.4), and after 60 min of osmification with a 2% OsO_4_ solution (in distilled water, pH 7.4) and repeated rinsing in distilled water for 10 min, the samples were dehydrated through a graded series of ethanol (from 50% to 100%, for 10 min in each concentration) and proceeded through propylene oxide [58,59]. After dehydration, the samples were embedded in an epoxy-based plastic (Durcupan ACM; Sigma) and polymerized at 56 °C for 48 h [58]. Amounts of 50 nm of ultrathin sections were cut from the plastic blocks with either an Ultracut UCT ultramicrotome (Leica; Wetzlar, Germany) or an RMC Boeckler PTPCZ Ultramicrotome (RMC Boeckler, Boeckeler Instruments, Inc., Tucson, AZ, USA). The ultrathin sections were then placed on single-hole formvar-coated copper grids (Electron Microscopy Sciences; Hatfield, PA, USA), contrasted with 2% uranyl acetate (Electron Microscopy Sciences) in 50% ethanol (Molar) and 2% lead citrate (Electron Microscopy Sciences) in distilled water (Reynolds, 1963; Hayat, 1970). The specimens were then screened with two separate microscope labs using two transmission electron microscopes: a JEM-1400Flash (JEOL; Tokyo, Japan) and a Delong LVEM 25 (Delong Instruments a.s., Brno, Czech Republic).

### 4.4. Electron Microscopic Tomography

For electron tomography, samples prepared as previously described on 150 square mash grids were placed in a high-tilt EM holder head (JEOL; Tokyo, Japan); then areas of interest suitable for tomography were selected. A single image from each angle was recorded with Recorder for TEM (System in Frontier; Tokyo, Japan) in the range of −44 to +44 tilting angles. Manual focus and automatic position tracking were used along with the whole tilt series. Image batch files were saved to tmg file format for further reconstruction, where pictures were built into an aligned image block using Composer (System in Frontier). After selecting the area of interest for prealignment, fiducial ultrastructure, like edges and areas with high contrast differences, were marked; then cross-correlation alignment and sinogram-based filtered back-projection were performed. A reconstructed block exported as an inverse black-and-white bin file was processed in Visualizer-Kai (System in Frontier). Min, max, gamma, and alpha values were set in gradient mode for the best visualization on the reconstructed opaque map. All of the visualized regions (cell area, full particle areas, spike proteins, and nucleocapsid) were segmented manually in the ROI subcomponent of Visualizer-Kai, saved to new volumes, and colored using single-color maps for better visualization.

### 4.5. Flow Cytometry Analysis of SDC Expression

The SDC expression of the applied cell lines (K562 cells, SDC4 transfectants, L929, and RAW 264.7) was measured with flow cytometry by using APC-labeled anti-human SDC4 (R&D Systems, Minneapolis, MN, USA; SDC4: monoclonal rat IgG2a clone #336304, cat. no. FAB29181A) or primary anti-murine SDC antibodies (mouse SDC1, cat. no. AF3190, R&D Systems, Minneapolis, MN, USA; mouse SDC2, cat. no. PA5-95938, Invitrogen, Waltham, MA, USA; mouse SDC3, cat. no. PA5-47377, Invitrogen, Waltham, MA, USA; mouse SDC4-APC labeled antibody, cat. no. 130-109-831, Miltenyi Biotec, Bergisch Gladbach, Germany) and appropriate secondary antibodies (rabbit anti-goat IgG (H+L) cross-adsorbed secondary antibody, Alexa Fluor™ 633, cat. no. A-21086, Invitrogen, Waltham, MA, USA; goat anti-rabbit IgG (H+L) cross-adsorbed secondary antibody, Alexa Fluor™ 633, cat. no. A-21070, Invitrogen, Waltham, MA, USA) and respective isotype controls according to the manufacturer’s protocol.

### 4.6. Flow Cytometry Analysis of SARS-CoV-2 Uptake

WT K562, L929, RAW 264.7, and SDC4 transfectants were utilized to quantify the internalization of inactivated SARS-CoV-2. Briefly, 6 × 10^5^ cells/mL in DMEM/F12 medium were incubated with SARS-CoV-2 (at 1 MOI) for 3 h at 37 °C. After 3 h of incubation, the cells were trypsinized (with the method described by Nakase et al. [60,61]) to remove the extracellularly attached virus particles from the cell surface. Then the cells were washed, fixed, permeabilized, and blocked with the appropriate serum for 1 h at room temperature. The cells were then treated with mouse monoclonal (1A9) to SARS spike glycoprotein (Abcam, Cambridge, UK, cat. no. 273433), followed by treatment with AF 488-labeled goat anti-mouse IgG (Invitrogen, Carlsbad, CA, USA, cat. no. A-11001). The samples were then rinsed three times with PBS containing 1% BSA and 0.1% Triton X-100 and progressed towards flow cytometry. Cellular uptake was then measured by flow cytometry using an Amnis FlowSight imaging flow cytometer (Amnis Corporation, Seattle, WA, USA). A minimum of 5000 events per sample were analyzed. Appropriate gating in a forward-scatter-against-side-scatter plot was utilized to exclude cellular debris and aggregates. Fluorescence analysis was conducted with the Amnis IDEAS analysis software.

### 4.7. Flow Cytometry Analysis of SARS-CoV-2 Uptake

SDC4 knockdown (KD) in L929 and RAW cells was performed using a lentiviral vector system specific to mouse SDC4 shRNA (cat. no. sc-36589-SH) according to the manufacturer’s protocol (Santa Cruz Biotechnology, Inc., Dallas, TX, USA). Stable KD cells were selected in 2 mg G418 and sorted using imaging flow cytometry (Amnis FlowSight, Luminex Corporation, Austin, TX, USA) with anti-SDC4 antibody and respective isotype control, as described above. The cellular expression of SDC4 following knockdown was also determined with Western blotting, as described previously [62].

### 4.8. Confocal Microscopy of In Vitro Colocalization

The colocalization of SARS-CoV-2 with SDC4 in L929 and RAW 264.7 cells was visualized by confocal microscopy. L929 and RAW 264.7 cells were grown on poly-D-lysine-coated glass-bottom 35 mm culture dishes (MatTek Corp., Ashland, MA, USA). After 24 h, the cells were incubated with SARS-CoV-2 (1 MOI) for 3 h at 37 °C. The cells were then rinsed two times with ice-cold PBS and fixed in 4% paraformaldehyde (Sigma, St. Louis, MO, USA), the cell membranes were permeabilized (0.1% Triton X-100), and the cells were blocked with the appropriate serum for 1 h at room temperature, followed by the specific spike S1 and SDC4 antibody treatments as described for the flow cytometry analyses. The samples were then rinsed three times with PBS containing 0.1% Triton X-100, then stained with DAPI (1:5000) for 15 min, washed three times with PBS, and embedded in Fluoromount G (SouthernBiotech, Birmingham, AL, USA) [20,54]. The fluorescence distribution was then analyzed on a Leica DMi8 microscope equipped with an Aurox Clarity laser-free confocal unit. Sections presented were taken approximately at the midheight level of the cells. Photomultiplier gain and illumination power were identical within each experiment. The Aurox Visionary software was used for image acquisition by confocal microscopy. For colocalization analyses, the images were analyzed with ImageJ’s (NIH, Bethesda, MD, USA) JACoP plugin.

### 4.9. Cell Viability Measurements

The effect of SARS-CoV-2 and spike on cell viability was assessed with the EZ4U cell proliferation assay (Biomedica GmBH, Vienna, Austria, cat. no. BI-5000) according to the instructions of the manufacturer. Absorbance was measured with a BioTek Cytation 3 multimode microplate reader.

### 4.10. Animal Experiments

2 × 10^6^ inactivated SARS-CoV-2 particles in 200 uL of PBS (Lonza, Basel, Switzerland; cat. no. BE17-516F) were injected intravenously (i.v.) into 12-month-old C57BL/6 mice. Control mice received 200 uL pure PBS (i.e., containing no virus) injections i.v. Each group (i.e., the SARS-CoV-2 treated and the controls) were made of 6 animals. After 3 h of incubation, the mice were anesthetized with 2,2,2-tribromoethanol (cat. no. T48402, Merck KGaA, Darmstadt, Germany), and blood was collected with cardiac puncture. After transcardial perfusion with ice-cold PBS (2 mL/min, 8 min), the brain, the heart, the liver, the lung, and the spleen were removed and frozen in dry ice for further examinations.

### 4.11. Measuring Mouse Tissue Concentrations of SARS-CoV-2 Spike

Brain, heart, lung, liver, and spleen samples were homogenized in lysis buffer (Qiagen) in 1% NP-40/PBS in a Complete Mini EDTA-free protease inhibitor cocktail (Roche). Tissue lysates were analyzed with a RayBio^®^ COVID-19/SARS-CoV-2 Spike Protein ELISA Kit (cat. no. ELV-COVID19S2, RayBiotech, Inc., Peachtree Corners, GA, USA) according to the manufacturer’s instructions.

### 4.12. Immunohistochemistry of Mouse Tissue Samples

For immunohistochemistry, mouse tissue samples (*n* = 6 mice per group) were fixed for 18 h in 4% paraformaldehyde (cat. no. P6148, Sigma-Aldrich), then dehydrated in an ethanol series, cleared with xylene (cat. no. 00699464, Avantor Inc., Radnor, PA, USA), and embedded in paraffin (cat. no. 26154.291, Avantor Inc.). Amount of 10 μm of thick sections were finally cut with a microtome (Leica Biosystems Inc., Buffalo Grove, IL, USA), and sections were collected on SuperFrost Plus^®^ slides (Thermo Fisher Scientific Inc., Waltham, MA, USA). Antigen detection was carried out with heat-induced antigen recovery. Slides were first immersed in citrate buffer heated to 95–100 degrees for 10 min, then cooled to room temperature for about 20 min. Next, the slides were placed in a blocking solution (5% goat or donkey serum diluted in 0.1% PBST) at room temperature for 30 min, and then the blocking solution was removed without rinsing. The slides were then incubated with primary antibodies (mouse monoclonal, 1A9, to SARS spike glycoprotein, Abcam, Cambridge, UK, cat. no. 273433) and APC-labeled anti-murine SDC4 antibodies (cat. no. 130-109-831, Miltenyi Biotec, Bergisch Gladbach, Germany), diluted in a blocking solution (1% BSA or goat serum in 0.1% PBST) at room temperature for 1 h or at 4 °C overnight. Slides were then rinsed with PBST three times for 10 min each at room temperature, followed by staining with 100 uL of AF 488-labeled goat anti-mouse IgG (Invitrogen, Carlsbad, CA, USA, cat. no. A-11001) diluted in a blocking solution for 1 h at room temperature. The slides were then rinsed three times with PBS at room temperature for 10 min and, using mounting media, were covered with cover plates. For colocalization analyses, the images were analyzed with ImageJ’s (NIH, Bethesda, MD, USA) JACoP plugin [18].

### 4.13. Measuring TNF-α Mouse Tissue Concentrations

Tissue samples were homogenized in lysis buffer (cat. no. 79216, Qiagen, Düsseldorf, Germany) in 1% NP-40/PBS in a Complete Mini EDTA-free protease inhibitor cocktail (cat. no. 11836170001, Roche, Basel, Switzerland), and tissue lysates were analyzed with a mouse TNF-alpha Quantikine ELISA Kit (cat. no. MTA00B, R&D Systems, Minneapolis, MN, USA) according to the manufacturer’s instructions.

### 4.14. Statistical Analysis

Results are expressed as means + standard error of the mean (SEM). Differences between experimental groups were evaluated by using one-way analysis of variance (ANOVA). Values of *p* < 0.05 were accepted as significant [20,54].

## Data Availability

Data are contained within the article or Supplementary Materials.

## Supplementary Materials

The following supporting information can be downloaded at: https://www.mdpi.com/article/10.3390/ijms23147609/s1.
