# Supplementary material for: Biodistribution and Cellular Internalization of Inactivated SARS-CoV-2 in Wild-Type Mice
Source: Int J Mol Sci. 2022 Jul 9;23(14):7609. doi: 10.3390/ijms23147609 (PMC9316427; doi:10.3390/ijms23147609)
Supplement: Supplementary file 1 [file ijms-23-07609-s001.zip › ijms-1776322-supplementary.pdf]

Supplementary Materials: Biodistribution and cellular internalization of inactivated SARS-CoV-2 in wild-type mice

Anett Hudák, Gareth Morgan, Jaromir Bacovsky, Roland Patai, Tamás F. Polgár, Annamária Letoha, Aladar Pettko-Szandtner, Csaba Vizler, László Szilák, and Tamás Letoha

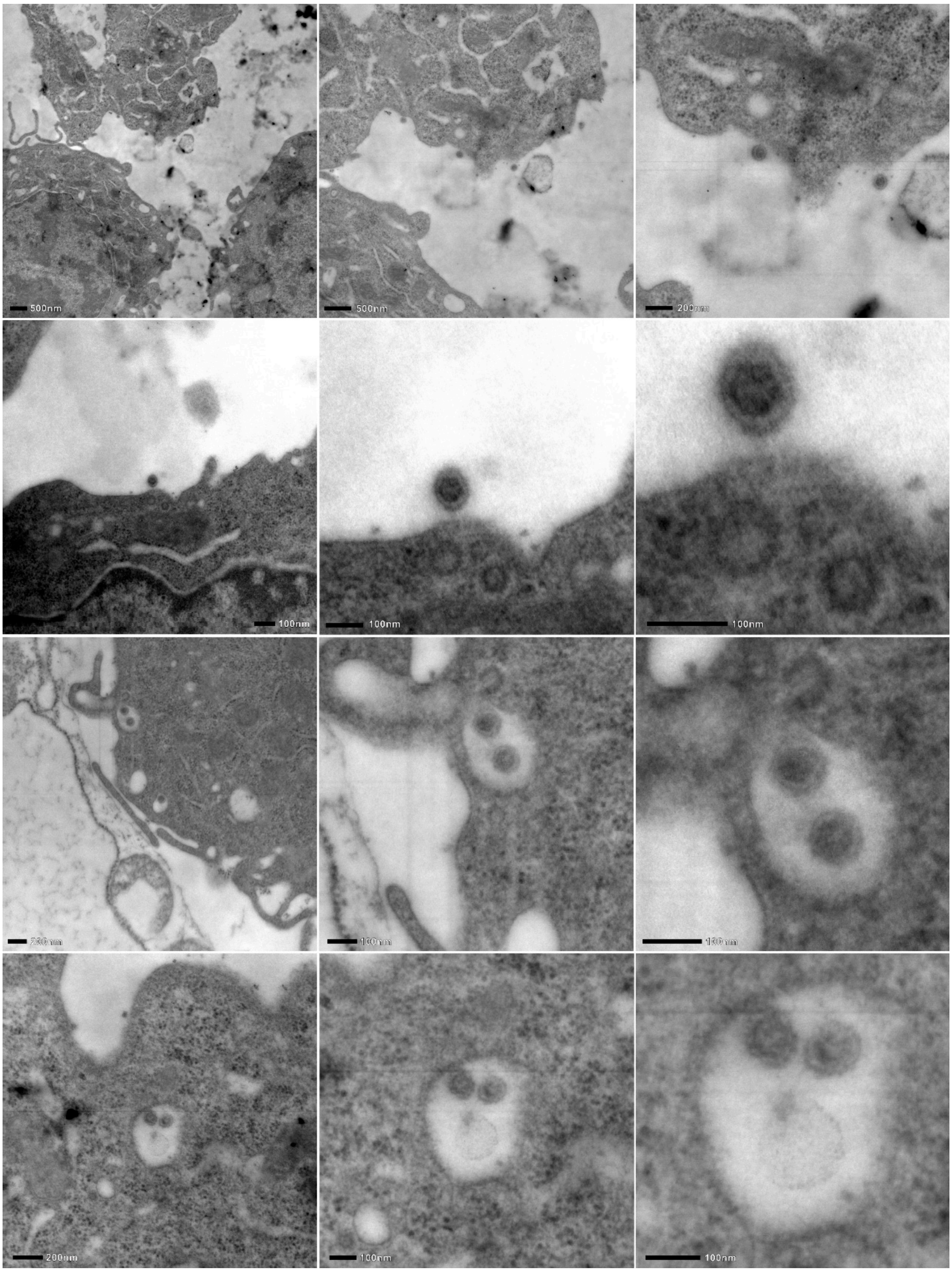

Figure S1. Electron microscopic visualization of SARS-CoV-2 internalization into RAW cells. RAW cells were incubated with heat-inactivated SARS-CoV-2 (at 1 MOI) for various amounts of time (10, 30, or 180 min) at 37 °C. After incubation, the cells were washed, fixed, and SARS-CoV-2 internalization was analyzed with a Delong LVEM 25 electron microscope. Representative images of three independent experiments are shown.

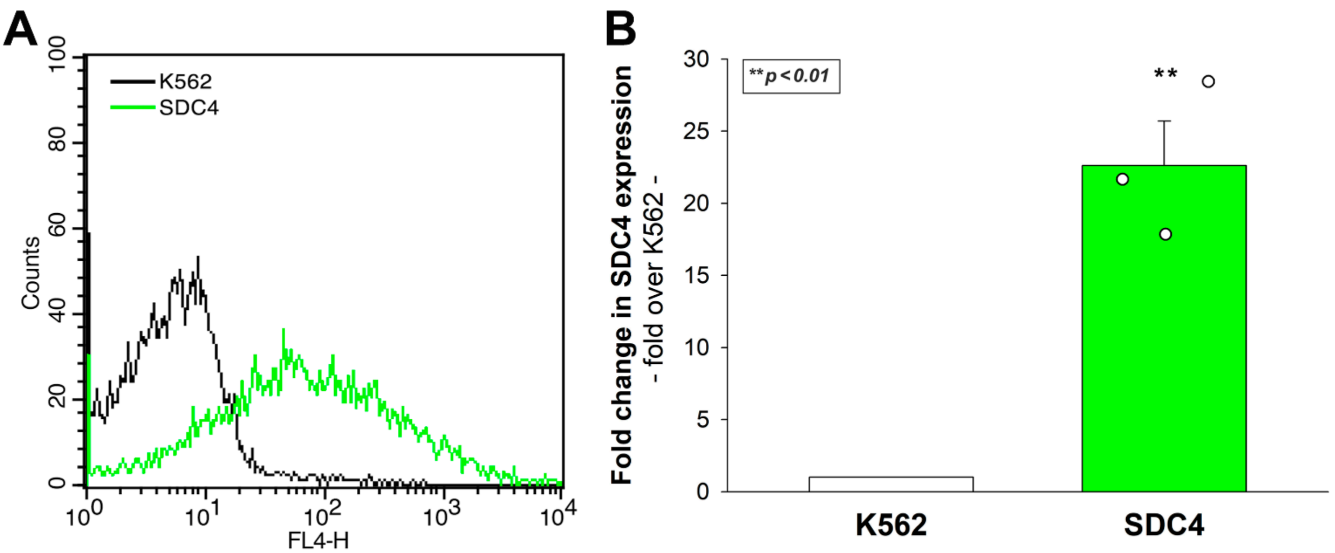

Figure S2. SDC4 expression of K562 cells and SDC4 transfectants. Stable SDC transfectants created in wild-type (WT) K562 cells were selected by measuring SDC4 expression with flow cytometry (Becton Dickinson FACScan) using APC-labeled anti-SDC4 antibodies. **A**: Representative flow cytometry histograms showing the SDC4 expression of SDC4 transfectants and WT K562 cells. **B**: Detected SDC4 levels were normalized to WT K562 cells as standards. The bars represent the mean + SEM of three independent experiments. Statistical significance vs. WT K562 cells (standards) was assessed with analysis of variance (ANOVA). ** $p < 0.01$.

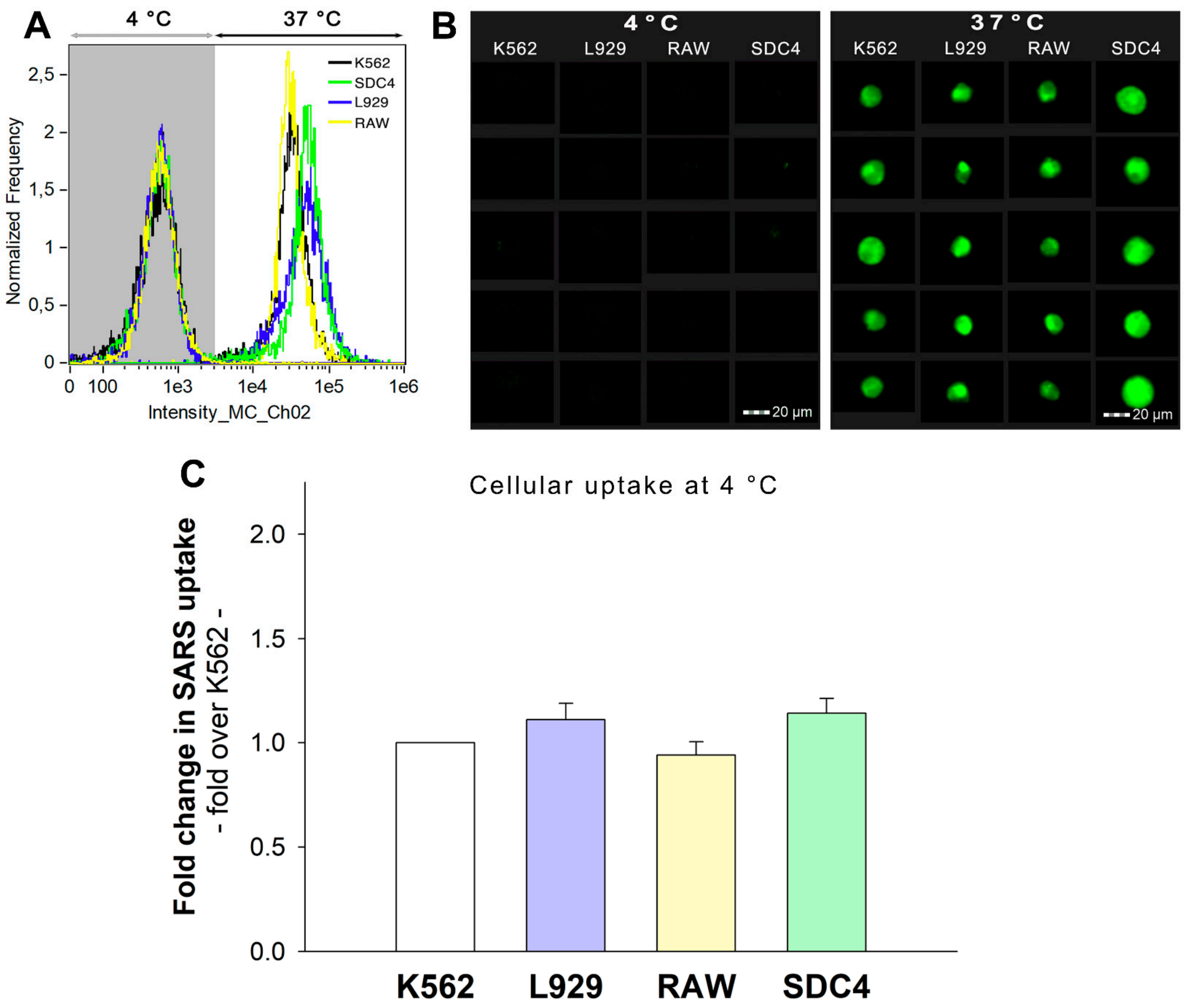

Figure S3. Flow cytometric assessment of SARS-CoV-2 uptake in human and murine cell lines at 4°C. L929, RAW, K562 cells, and SDC4 transfectants (created in K562 cells) were incubated with heat-inactivated SARS-CoV-2 (at 1 MOI) for 3 h at 4 °C. After incubation, the cells were washed, trypsinized, fixed, permeabilized, and treated with antibodies specific for the spike glycoprotein (along with AF 488-labeled secondary antibodies). Cellular uptake of SARS-CoV-2 was then analyzed with imaging flow cytometry. **A,B**: Representative flow cytometry histograms and fluorescent images showing the intracellular fluorescence of SARS-CoV-2-treated cells at 4 and 37 °C. **C**: Fluorescence intensities detected in cells treated with inactivated SARS-CoV-2 at 4 °C were normalized to SARS-CoV-2-treated K562 cells as standards. The bars represent the mean + SEM of three independent experiments. Statistical significance vs. standards was assessed with ANOVA. Compared to K562 cells (i.e., standards), no statistically significant differences were detected in the viability of SARS-CoV-2-treated cells.

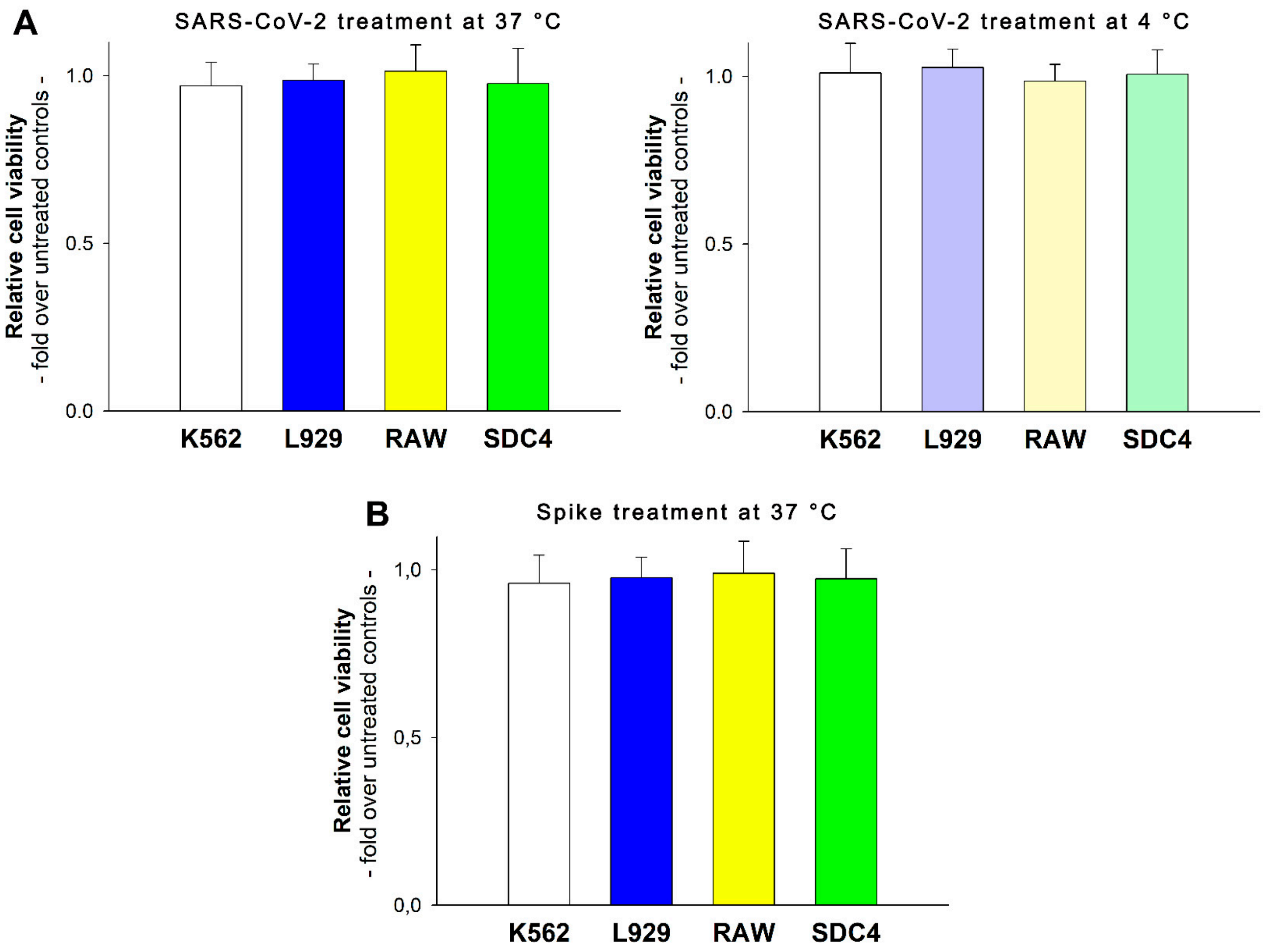

Figure S4. Inactivated SARS-CoV-2 and spike does not affect the cellular viability. **A,B**: L929, RAW, K562 cells, and SDC4 transfectants (created in K562 cells) were incubated with heat-inactivated SARS-CoV-2 (at 1 MOI) for 3 h at 37 or 4 °C (**A**) or SARS-CoV-2 spike protein (50 nM) for 3 h at 37 °C (**B**). Cellular viability was then measured with EZ4U assay and detected measures were then normalized to untreated cells as controls. The bars represent the mean + SEM of three independent experiments. Statistical significance vs. controls was assessed with ANOVA. Compared to controls, no statistically significant differences were detected in the viability of SARS-CoV-2-treated cells.

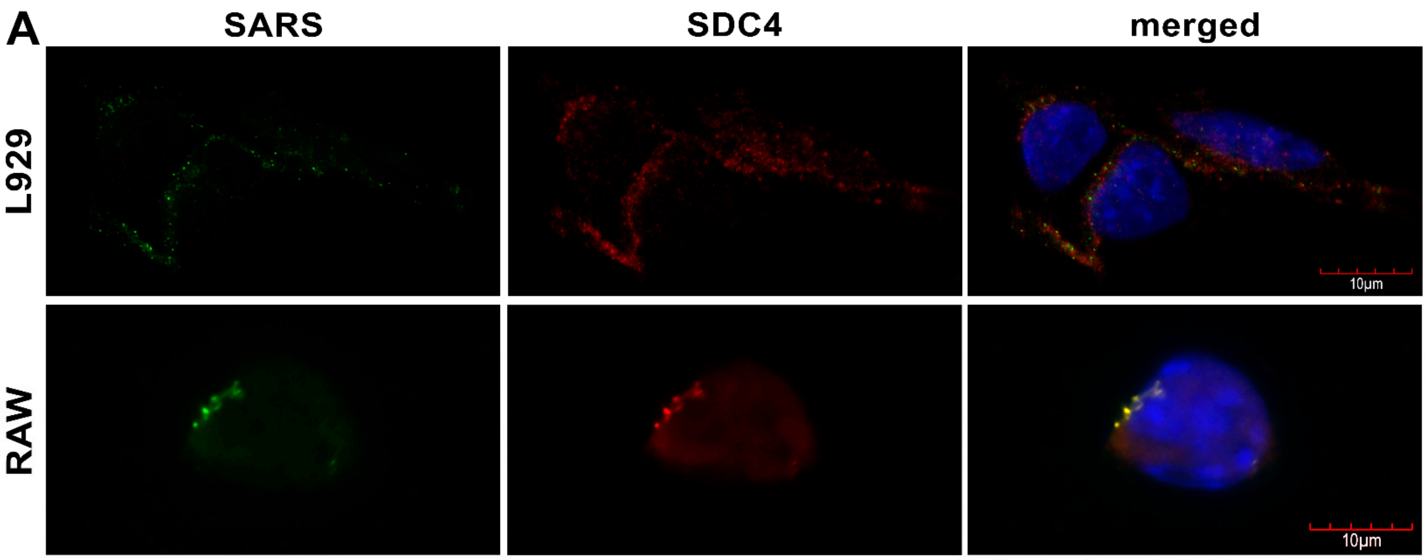

B	Mander's overlap coefficient (MOC)	Pearson correlation coefficient (PCC)
L929	0.70 ± 0.02	0.72 ± 0.03
RAW	0.75 ± 0.02	0.79 ± 0.02

Figure S5. SARS-CoV-2 colocalizes with SDC4 on the cell membrane. L929 and RAW cells were incubated with heat-inactivated SARS-CoV-2 (at 1 MOI) for 3 h at 4 °C. After incubation, the cells were washed, fixed, and treated with antibodies specific for the spike glycoprotein (AF 488-labeled) and SDC4 (APC-labeled). Colocalization of SARS-CoV-2 with SDCs was analyzed with confocal microscopy. **A**: Microscopic analyses of SARS-CoV-2 and SDC colocalization. Representative images of three independent experiments are shown. Scale bar = 10 µm. **B**: The MOC and PCC ± SEM for the overlap and colocalization of SDC with SARS-CoV-2 (indicated below the images) were calculated by analyzing 18 images. Scale bar = 10 µm.

o

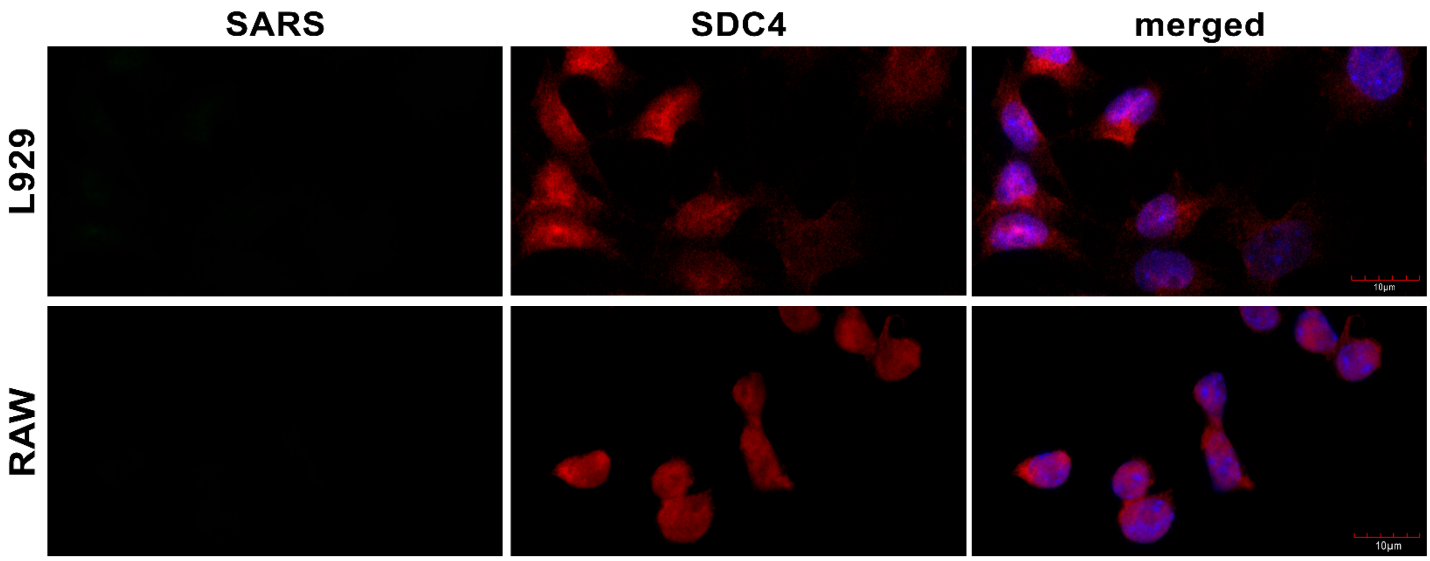

Figure S6. Microscopic studies on SARS-CoV-2-untreated control cells (i.e., those receiving no SARS-CoV-2 treatment). L929 and RAW cells untreated with SARS-CoV-2 washed, fixed, permeabilized, and treated with antibodies specific for the spike glycoprotein (and AF 488-labeled secondary antibody) and SDC4 (APC-labeled). Representative images of three independent experiments are shown. Scale bar = 10 µm.

Table S1. SDC4 expression of various tissues as presented in the Human Protein Atlas

	**Protein expression**	**RNA expression** [1]
**Brain**	High	Medium (nTMP: 110.7)
**Heart**	Medium	Medium (nTMP: 66.4)
**Liver**	Medium	High (nTMP: 414.4)
**Lung**	High	Medium (nTMP: 169.8)
**Spleen**	Low	Low (nTMP: 32.6)

[1] The RNA-seq tissue data is reported as nTPM (normalized protein-coding transcripts per million), corresponding to mean values of the different individual samples from each tissue.

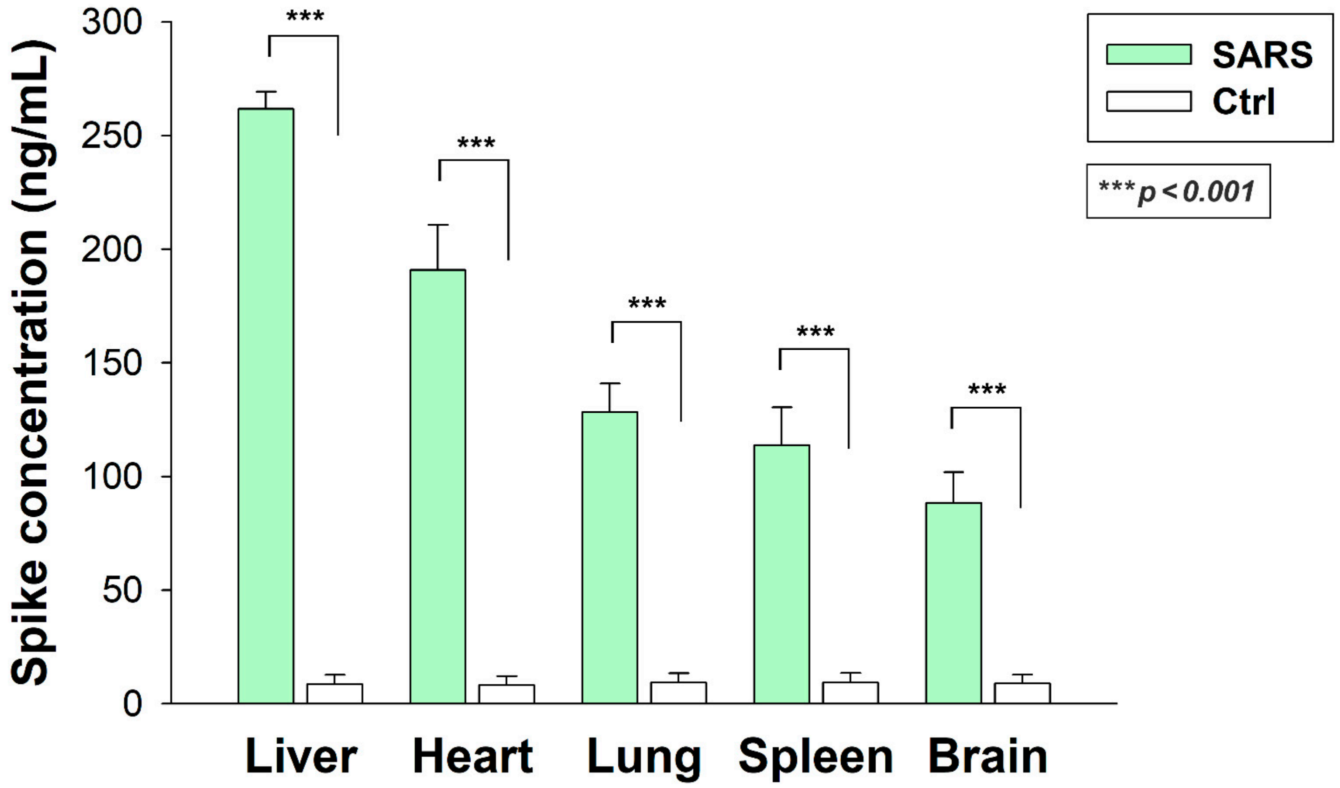

Figure S7. SARS-CoV-2 spike concentration in SARS-CoV-2-treated mice (SARS) and untreated controls (Ctrl) measured with a spike-specific ELISA kit. Each group contained 6 animals. The bars represent the mean + SEM. Statistical significance vs controls was assessed with ANOVA. *** $p < 0.001$.

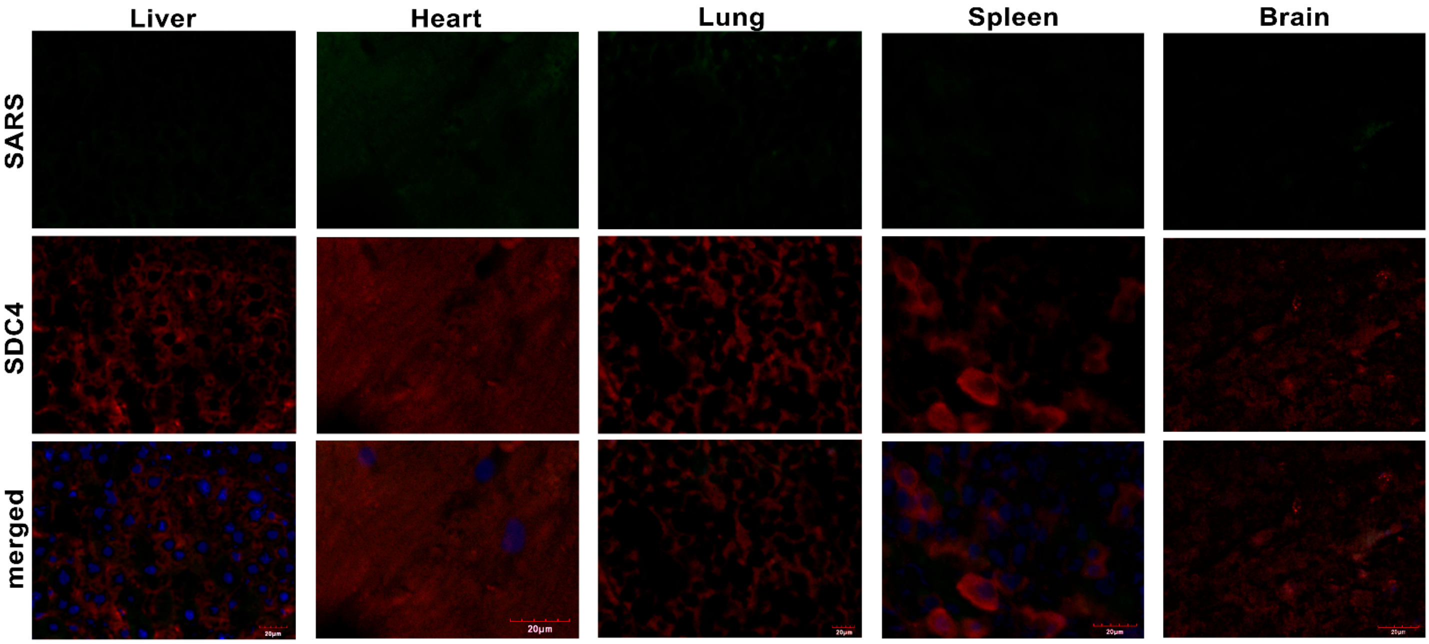

Figure S8. Microscopic studies on SARS-CoV-2-untreated control mice (i.e., those receiving no SARS-CoV-2 treatment). Liver, heart, lung, spleen and brain samples of mice receiving i.v. administration of 200 µL of PBS were treated with antibodies specific SARS-CoV-2 spike (AF488-labeled) and SDC4 (APC-labeled). Representative images of three independent samples of six control animals are shown. Scale bar = 20 µm.
